# Non-Stationary Non-Hermitian “Wrong-Sign” Quantum Oscillators and Their Meaningful Physical Interpretation

**DOI:** 10.3390/e25040692

**Published:** 2023-04-19

**Authors:** Miloslav Znojil

**Affiliations:** 1 Department of Physics, Faculty of Science, University of Hradec Králové, Rokitanského 62, 500 03 Hradec Králové, Czech Republic; znojil@ujf.cas.cz; 2 The Czech Academy of Sciences, Nuclear Physics Institute, Hlavní 130, 250 68 Řež, Czech Republic

**Keywords:** quantum mechanics of non-stationary unitary systems, non-Hermitian interaction representation, quasi-Hermitian form of the observables, a complete set of evolution equations, internal consistency of the probabilistic interpretation, application to asymptotically repulsive potential models

## Abstract

In the framework of quantum mechanics using quasi-Hermitian operators the standard unitary evolution of a non-stationary but still closed quantum system is only properly described in the non-Hermitian interaction picture (NIP). In this formulation of the theory both the states and the observables vary with time. A few aspects of implementation of this picture are illustrated via the “wrong-sign” quartic oscillators. It is shown that in contrast to the widespread belief, both of the related Schrödinger-equation generators G(t) and the Heisenberg-equation generators Σ(t) are just auxiliary concepts. Their spectra are phenomenologically irrelevant and, in general, complex. It is argued that only the sum H(t)=G(t)+Σ(t) of the latter operators retains the standard physical meaning of the instantaneous energy of the unitary quantum system in question.

## 1. Introduction

In the d—dimensional and centrally symmetric anharmonic oscillator quantum Hamiltonian

(1) $$h^{\left(AHO\right)}\left(\lambda \right)=-\frac{1}{2}\;\Delta +\frac{1}{2}{\;|x|}^{2}+\lambda^{2}\;{\left|x\right|}^{4}\;,\;\;\;\;\;x\in R^{d}$$

the “correct” choice of the sign at the asymptotically dominant and confining $O(|x{|}^{4})$ component of the interaction is known to be responsible for the fact that the corresponding energy levels $E_{n}\left(\lambda ,\ell \right)$ form a discrete, real, and positive set where n,ℓ=0,1,…, with *ℓ* being the angular-momentum quantum number [1].

Incidentally, it is less widely known that the *same* discrete spectrum can be also interpreted as corresponding to a very different complex, ℓ—dependent and, first of all, “wrong-sign” form of the one-dimensional and asymptotically quartic (and, obviously, manifestly non-Hermitian) Hamiltonian-like operator of Theorem Nr. 1 in paper [2],

(2) $$\tilde{Q}\left(i\lambda ,j\right)=-\frac{d^{2}}{dx^{2}}+\frac{j}{2}-ij\lambda \;x+x^{2}-2i\lambda \;x^{3}-\lambda^{2}\;x^{4}\;,\;\;\;\;\;j=2\ell +d-2\;.$$

The authors of the latter paper interpreted their isospectrality result as a mere mathematical curiosity [3]. Nevertheless, a few years later, the perception of the wrong-sign models of the type (2) has changed: at present, it is widely accepted that there exist many complex and non-Hermitian Hamiltonians $H\neq H^{†}$ possessing the real and discrete, bound-state-mimicking spectra [4,5,6].

In retrospect, one can identify two decisive ideas behind the acceptability and current acceptance of the latter class of counterintuitive models in the quantum model-building practice. The emergence of the first idea dates back to the brief letter [7] in which Bender with Turbiner proposed that aside from the conventional ordinary differential operators $H=-d^{2}/dx^{2}+V\left(x\right)$ in which the variable *x* is real, a consistent quantum theory might be also obtained when one performs an analytic continuation of the model to a suitable complex curve of x∈C∉R.

This idea was fully developed and presented, in 1998, in a truly influential letter by Bender and Boettcher [8]. These authors proposed that after analytic continuation (during which *x* becomes complex and loses its observability status in general), the ordinary differential operator $H=-d^{2}/dx^{2}+V\left(x\right)$ might still be accepted as a consistent physical Hamiltonian yielding the conventional, real, and observable bound-state energy spectrum (see also review [4]).

In the language of mathematics this means that one has to work, in general, with a “complex coordinate”, *x*. A few examples of such a choice are outlined in Section 2 below. In this context, the second, equally important support of the phenomenological acceptability of the ordinary differential models defined along the complex, “unphysical” curve of *x* has been found in the “quasi-Hermitian” reformulation of the standard quantum mechanics, as provided by Scholtz et al. [9]. These authors, in their own words, “established a general criterion for a set of non-Hermitian operators to constitute a consistent quantum mechanical system”, which would “allow for the normal quantum-mechanical interpretation” and which would, in the usual Hilbert space $L^{2}\left(C\right)$, “involve the construction of a metric”, i.e., in our present notation, of a suitable nontrivial and, in general, Hamiltonian-dependent operator Θ=Θ(H)≠I.

A more explicit account of the resulting amended representation of the stationary quantum systems (i.e., of the theory which we will call non-Hermitian Schrödinger picture, NSP) may be found outlined in Section 3 below. In its applications, the analytic continuation mathematics appeared to yield many new and unusual eligible non-Hermitian, but time-independent, Hamiltonians H≠H(t) with real spectra (cf. the review chapters in monograph [10]).

Whenever people have managed to construct the metric (which appeared to be the case, in particular, for the wrong-sign oscillators of Section 2 below), the new models appeared to be also endowed with a meaningful physical (i.e., standard probabilistic) interpretation. Naturally, the restriction of the above-mentioned NSP constructions to the stationary wrong-sign potentials is to be considered rather severe. For this reason, Fring and Tenney [11] posed the question of what happens when these potentials become manifestly time-dependent.

In their search for inspiration, Fring and Tenney restricted their attention to several stationary wrong-sign oscillators (this means, to the models listed and discussed here in Section 2). Having proposed a manifestly time-dependent generalization of these models, they arrived at the highly nontrivial and encouraging conclusion that in spite of an enormous increase of the complexity of the formalism (to be called here non-Hermitian interaction picture, NIP), they managed to reach their ultimate goal of the closed-form construction of all of the relevant operators.

In our present paper we intend to complement the Fring’s and Tenney’s non-stationary and non-Hermitian wrong-sign oscillator results with a few important methodical addenda. As we already partially indicated, our message will be preceded by a compact review of the stationary wrong-sign models of interest (in Section 2) and by a more detailed description and discussion of their physical meaning in Section 3. Then, the core of our message will be presented in the methodically oriented Section 4 (where we outline the basic features of the underlying NIP theory) and in the application-oriented Section 5, where the Fring’s and Tenney’s construction will be recalled and complemented by an outline of an alternative approach.

A discussion and summary of our results will finally be added in Section 6.

## 2. Complex Potentials with Real Energy Levels

For a given, preselected, and, importantly, stationary and non-Hermitian Hamiltonian *H* with the real spectrum and with properties H≠H(t) and $H\neq H^{†}$ in an unphysical, auxiliary Hilbert space $L^{2}\left(C\right)$ *alias* $H_{auxiliary}$, the assignment of an alternative, amended physical Hilbert space (endowed with a nontrivial ad hoc metric Θ) is of decisive importance. It makes such an operator quasi-Hermitian [12],

(3) $$H^{†}\;\Theta =\Theta \;H\;,$$

i.e., self-adjoint with respect to the amended metric Θ≠I, i.e., self-adjoint in an amended, “correct” Hilbert space $L^{2}\left(C,\Theta \right)$ *alias* $H_{physical}$.

In such a context, we felt motivated by the recent developments of the field in which the stationary-metric formalism has been replaced by its highly nontrivial non-stationary metric generalization (to be called non-Hermitian interaction picture, NIP, in what follows). In such an innovative framework, even the “anomalous” potentials as sampled in Equation (2) might be assigned a meaningful quantum-theoretical interpretation using the theory described, e.g., in the comprehensive reviews [4,5,6].

### 2.1. Constructions Using Perturbation Theory

In our present paper, we are going to pay detailed attention to the possibility of a consistent and meaningful quantum-theoretical interpretation of the complex and manifestly non-Hermitian Hamiltonians with the real bound-state spectra, as demonstrated by the wrong-sign example. Our analysis will be restricted just to a few unitary quantum models using the wrong-sign potentials for which the wave-function solutions of the corresponding ordinary differential Schrödinger equations may be expected not to contradict a conventional bound-state interpretation.

A consistent realization of the latter possibility can rely upon the existing theory [4,5,6,9]. Temporarily, we will assume that all of our Hamiltonians of interest remain stationary, H≠H(t). This will enable us to explain, more easily, that such a technical restriction simplifies the meaningful physical interpretation of the systems and leads to a simple ad hoc reformulation of quantum mechanics called non-Hermitian or, better, quasi-Hermitian [12] quantum mechanics.

Initially, our interest in the problem was motivated by the current textbooks on quantum mechanics in which one can find multiple illustrative examples of the bound state energy spectra of a particle moving in a real, local, and confining potential $V(\vec{x})$. Aside from the exactly solvable one-dimensional or centrally symmetric harmonic oscillator $V^{\left(HO\right)}\left(\vec{x}\right)={\left|\vec{x}\right|}^{2}$, extensive attention is being paid also to its anharmonic and, in particular, quartic perturbations. Although the meaningful physical interpretation of the states in the asymptotically confining (i.e., “correct-sign”) version $V_{\lambda }^{\left(AHO\right)}\left(\vec{x}\right)=|\vec{x}{|}^{2}+\lambda^{2}\;{\left|\vec{x}\right|}^{4}$ of the potential remains standard, unexpected problems emerge when one tries to construct the energy levels $E_{n}\left(\lambda ,\ell \right)$ using the Rayleigh-Schrödinger perturbation series in the powers of λ.

During the history of such an approach as summarized briefly in [2], the problems caused by the divergence of perturbation series were resolved (or, better, efficiently circumvented) due to its Borel summability. For our present purposes it is important that as a byproduct of these results, a way is found to open toward a fully rigorous mathematical understanding of the spectra of the wrong-sign operators as sampled in Equation (2) above.

Initially, and not too surprisingly, people did not call the corresponding Hamiltonian-like operators Hamiltonians. One of the reasons was that the normalizability of its eigenstates appeared to be merely guaranteed by a fine-tuned interference between the separate components of the asymptotically repulsive “potential”. Thus, people were only speaking, in such a case, about “unstable oscillators” [2].

The paradigm was only changed when Bender with Boettcher [8] turned attention to the undeniable relevance of the parity times time reversal *alias* PT—operator symmetry of the models with real spectra. On these grounds they decided to advocate for the anomalous non-Hermitian models with real spectra as, in some sense, phenomenologically acceptable.

### 2.2. Constructions Using PT—Symmetry

In traditional textbooks on quantum mechanics, the observables are assumed to be self-adjoint (see, e.g., [13]). Precisely this constraint has been criticized by Bender and Boettcher as too formal [8]. These authors initiated the development of an innovative, but still conceptually tenable, quantum theory (a.k.a. PT—symmetric quantum mechanics, PTQM, see, e.g., its detailed review [4]). In this approach (which may also be called non-Hermitian Schrödinger picture, NSP) the bound states were conjectured to be represented by the eigenvectors $|\psi \left(t\right)\rangle \in H^{\left(BB\right)}$ of an unusual, stationary (or quasi-stationary [5]), but manifestly non-Hermitian Hamiltonian $H\neq H^{†}$.

The impact of the Bender’s and Boettcher’s conjecture has been enhanced by their explicit demonstration that the hypothetical new family of the eligible non-Hermitian candidates for the quantum Hamiltonians of unitary systems may even contain a fairly rich subfamily of Hamiltonians with the conventional kinetic-energy plus potential-energy structure. In their explicit illustrative example, $H^{\left(BB\right)}=-d^{2}/dz^{2}+V\left(z\right)$, the potential has been chosen as complex but still local and analytic,

(4) $$V\left(z\right)=V^{\left(BB\right)}\left(z,\delta \right)=\lambda^{2}\;z^{2}\;{\left(iz\right)}^{\delta }\;,\;\;\;\;\delta \geq 0\;.$$

Strictly speaking, up to the exceptional harmonic oscillator special case with δ=0, the intuitive potential-energy treatment of such an interaction would not be appropriate. This is for two reasons. The first, less important one is that even the coupling constant is generally complex. Hence, unless δ=0,2,4,…, the interaction itself would not be too easily realizable in the laboratory, even when one succeeds in keeping the coordinate itself observable, z∈R.

The second counterintuitive feature of the PT—symmetric interaction (4) is that its definition admitting the real and observable coordinate z∈R *and* the real and observable bound-state energies $E_{0},E_{1},\ldots$ only appeared phenomenologically consistent in a finite interval 0≤δ<2 of the exponents. Otherwise, at negative δ>−1, a high-lying part of the energy spectrum has been found to be all complex (and, hence, unobservable) [8]. Subsequently, the whole spectrum becomes empty in the Herbst’s limit δ→ −1 [14]. In parallel, at any larger δ≥2, the correct asymptotic values of the variable *z* had to be chosen as complex and, hence, unobservable (cf., e.g., [8] or [15] for explanation).

### 2.3. The Jones and Mateo Wrong-Sign Model with δ=2

In the context of preceding paragraph a special role is played by Bender’s and Boettcher’s Hamiltonians with δ=2. A fairly persuasive illustration of the phenomenological, as well as formal appeal, of such a remarkable special case has been provided by Jones and Mateo [16]. In their analysis they considered the Hamiltonian

(5) $$H^{\left(JM\right)}\left(\lambda \right)=-\frac{d^{2}}{dz^{2}}-\lambda^{2}\;z^{4}\;$$

which is by far the simplest quartic wrong-sign oscillator with a real spectrum.

The Jones and Mateo proof of the reality of the spectrum of their model was constructive. First, they specified the explicit form of the complex contour of the “coordinate” *z*,

(6) $$z=z\left(x\right)=-2i\sqrt{1+ix}\;,\;\;\;\;\;x\in R\;.$$

In terms of the new real coordinate *x*, the original Hamiltonian (5) acquires the form of an ordinary differential operator $H=H_{0}+H_{1}$ (cf. Eq. Nr. (11) in loc. cit.) composed of a Hermitian part $H_{0}=p^{2}-p/2+16\lambda^{2}\left(x^{2}-1\right)$ and an anti-Hermitian component $H_{1}=i\left(xp^{2}+p^{2}\;x\right)/2-32i\lambda^{2}\;x$ (cf. Eq. Nr. (13) in loc. cit.).

Due to the simplicity of the Jones and Mateo wrong-sign model, it appeared feasible to prove that the “anomalous” operator $H^{\left(JM\right)}\left(\lambda \right)$ is in fact fairly well behaved and, first of all, strictly isospectral to another, conventional and self-adjoint Hamiltonian

(7) $${\tilde{h}}^{\left(JM\right)}\left(\lambda \right)=-\frac{d^{2}}{dy^{2}}-2\;\lambda \;y+4\;\lambda^{2}\;y^{4}\;.$$

**Figure 1:**
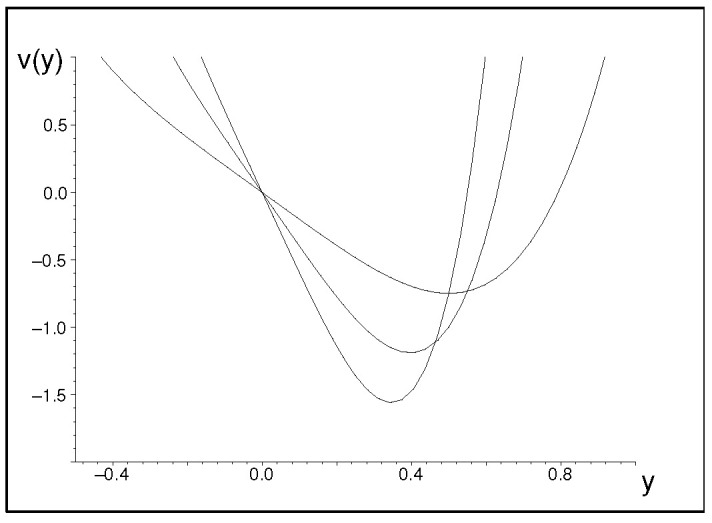
The shape of potential (8) at λ=1,2, and 3.

Here, the potential is safely confining and smooth (cf. its samples in Figure 1). Near its minimum $v\left(y_{\min}\right)=-3\lambda^{2/3}/4\;$ at $\;y_{\min}=1/\left(2\;\lambda^{1/3}\right)\;$ this potential is reasonably well approximated by the exactly solvable harmonic oscillator well,

(8) $$v\left(y\right)=-2\;\lambda \;y+4\;\lambda^{2}\;y^{4}\approx v\left(y_{\min}\right)+\omega^{2}{\left(y-y_{\min}\right)}^{2}+O\left[{\left(y-y_{\min}\right)}^{3}\right]\;,$$

with $\omega^{2}=v^{\prime \prime }\left(y_{\min}\right)/2=24\;\lambda^{2}\;y_{\min}^{2}$. With the growth of λ, the minimum decreases and the well becomes narrower. Despite the decrease of the minimum the value $E_{0}\approx v\left(y_{\min}\right)+\sqrt{6}\;\lambda^{2/3}$, the ground-state energy itself remains positive and growing because $\sqrt{6}-3/4>0$.

### 2.4. The Buslaev and Grecchi Anharmonic Wrong-Sign Model

In the above-mentioned perturbation theory-based analyses of the conventional “correct-sign” anharmonic oscillator bound-state energies $E_{n}\left(\lambda ,\ell \right)$, the mathematics become most interesting when one imagines that these energies can be represented by the Borel sum of the conventional (and, in this case, divergent) Rayleigh-Schrödinger perturbation series [1]. This opens the way toward the perception of the spectrum in the language of analytic functions.

In this spirit, Buslaev with Grecchi [2] recalled the the radial component of the conventional anharmonic oscillator Hamiltonian (1) and emphasized that after a rotation of the coupling in a complex plane (λ→iλ) one obtains a wrong-sign anharmonicity in infinity and a new type of singularity in the origin. After its regularization (via a complex shift of $r\to r_{\epsilon }\left(x\right)=x-i\epsilon$) they obtained a new Hamiltonian of the form

(9) $$H^{\left(BG\right)}=H_{\epsilon }\left(i\lambda ,j\right)=\frac{1}{2}\;\left(-\frac{d^{2}}{dx^{2}}+\frac{j^{2}-1}{4\;r_{\epsilon }^{2}\left(x\right)}+r_{\epsilon }^{2}\left(x\right)\right)-\lambda^{2}\;r_{\epsilon }^{4}\left(x\right)\;.$$

This operator is manifestly non-Hermitian but, in the Buslaev and Grecchi terminology, it is still TP—symmetric in $L^{2}\left(R\right)$, with *P* representing parity, and with *T* denoting the operator of complex conjugation (cf. remark Nr. 4 in [2]).

On this basis, the authors were able to prove that their oscillator (9) is isospectral with the fully conventional and safely self-adjoint and confining double-well Hamiltonian

(10) $$h^{\left(BG\right)}=Q\left(\lambda ,j\right)=-\frac{d^{2}}{dy^{2}}+j/2-j\;\lambda \;y+y^{2}-2\;\lambda \;y^{3}+\lambda^{2}\;y^{4}\;$$

(cf. Theorem Nr. 5 in loc. cit.). This implies that the shared spectrum of *both* of these Hamiltonians is real, discrete, and bounded from below.

One might conclude that in contrast to the widespread use of the strongly misleading nickname of “unstable oscillator” [11], the wrong-sign anharmonic oscillator (9) may be tentatively interpreted as representing a closed quantum system which is unitary and stable. Naturally, it would only be necessary to reformulate the conventional quantum mechanics of textbooks. Precisely such a reformulation of the abstract theory has been provided by Scholtz et al. [9]. Let us now recall and outline its basics.

## 3. Hidden Hermitian Theory in Stationary Regime

In many conventional introductions to quantum mechanics of unitary systems the states are represented by the time-dependent ket-vector elements |ψ(t)≻ of a suitable physical Hilbert space L while the operators q representing observables are usually kept time-independent, q≠q(t). Under these assumptions characterizing quantum theory formulated in the so-called Schrödinger picture (SP, ref. [13]) the predictions of the results of measurements are given in terms of matrix elements ≺ψ(t)|q|ψ(t)≻. Thus, in applications one only has to solve the Schrödinger equation

(11) $$i\frac{d}{dt}\;\left|\psi \left(t\right)\;\;≻\;\;=h\;\right|\psi \left(t\right)\;\;≻\;\;$$

while using the Hamiltonian which must be self-adjoint in L, $h=h^{†}$.

### 3.1. Non-Hermitian Schrödinger Representation

The situation becomes different when a candidate *H* for the Hamiltonian is non-Hermitian. Its standard quantum-mechanical interpretation can be then established in a way described, in 1992, by Scholtz et al. [9]. The basic idea of the reformulation of the quantum mechanics of unitary systems in a non-Hermitian Schrödinger picture (NSP, see [9] and also the more recent reviews [5,6]) can be seen in a replacement of the single physical Hilbert space of states (say, L) by the two mutually non-equivalent Hilbert spaces (say, $H_{physical}$ and $H_{auxiliary}$). In such a setting, one has to generalize the conventional Schrödinger picture (SP) and to replace the underlying usual Schrödinger Equation (11) with an appropriate modification.

The basic idea behind the generalization is that a state of the system (marked, say, by the Greek letter ψ and varying with time, ψ=ψ(t)) is perceived not only as a ket-vector element of the conventional textbook space L (to be written here as a “curly” ket |ψ(t)≻) but also as a (shared) element |ψ(t)〉 of both of the alternative Hilbert spaces $H_{physical/auxiliary}$ (with the state marked, in both spaces, by the same and most common version of the ket-symbol).

First of all, the formal SP–NSP equivalence of the description may be given the form of the one-to-one correspondence between the representations,

(12) $$|\psi^{\left(textbook\right)}\;\;≻\;\;=\Omega_{}\;|\psi^{\left(auxiliary\right)}\rangle \;.$$

Using the concept of the (by definition, nontrivial [9]) metric operator $\Theta =\Omega^{†}\;\Omega \neq I$, we may immediately introduce the bra vectors denoted as ≺ψ(t)| in L, as 〈ψ(t)| in $H_{auxiliary}$, and as 〈〈ψ(t)| in $H_{physical}$. Now, once we recall the mapping (12) we may simply avoid the related ambiguities via a reinterpretation of $H_{physical}$ as a mere modified version of space $H_{auxiliary}$ in which the inner product is amended,

(13) 〈ψ|χ〉→〈ψ|Θ|χ〉

or, equivalently, in which one simply represents 〈〈ψ(t)|=〈ψ(t)|Θ and |ψ(t)〉〉=Θ|ψ(t)〉.

One can notice that such a notation convention is compact, efficient, and consistent. For example, having an observable represented by an operator q in L (where it has to be self-adjoint), we can just work with its isospectral avatar in $H_{auxiliary}$,

(14) $$Q_{}=\Omega_{}^{\left(-1\right)}\;q_{}\;\Omega_{}\neq Q_{}^{†}\;.$$

Nevertheless, it is not necessary to emphasize the self-adjointness of $Q_{}$ in $H_{physical}$ because it is sufficient to stay working in $H_{auxiliary}$ where such a requirement acquires the metric-dependent form

(15) $$Q^{†}\;\Theta =\Theta \;Q\;.$$

This property of the operator *Q* imposed in the preferred but unphysical working Hilbert space $H_{auxiliary}$ is called, in mathematics, quasi-Hermiticity [9,12] *alias* Θ—pseudo-Hermiticity [5].

Using our present notation conventions we may summarize that the predictions mediated by the SP and NSP calculations are indistinguishable, in essence, due to the coincidence of matrix elements

(16) $$≺\;\;\psi^{}\left(t\right)\;|q_{}\;|\psi^{}\left(t\right)\;\;≻\;\;=\langle \;\langle \psi^{}\left(t\right)\;|Q_{}\;|\psi^{}\left(t\right)\rangle \;.$$

Thus, once we restrict our attention just to the NSP framework, it is sufficient to find a (time-independent) solution Θ=Θ(Q) for Equation (15). Then, for an evaluation of predictions (16) one just needs to know the time-dependent solution |ψ(t)〉 for the NSP evolution equation,

(17) $$i\frac{\partial }{\partial t}\;|\psi^{}\left(t\right)\rangle =H_{}\;|\psi^{}\left(t\right)\rangle \;.$$

Obviously, all of the calculations may be performed, exclusively, in the preferred “mathematical” Hilbert space $H_{auxiliary}$.

### 3.2. The Case of Stationary Wrong-Sign Potentials

The main problem addressed in the Jones and Mateo paper [16] was the construction of the inner-product metric $\Theta^{\left(JM\right)}$ which would make their wrong-sign quartic Hamiltonian (5) quasi-Hermitian in $H_{auxiliary}$. The essence of their discovery was that in the corresponding constraint (15) with $Q=H^{\left(JM\right)}\left(\lambda \right)$, viz., in relation

(18) $${\left[H^{\left(JM\right)}\right]}^{†}\left(\lambda \right)\;\Theta^{\left(JM\right)}=\Theta^{\left(JM\right)}\;H^{\left(JM\right)}\left(\lambda \right)$$

the insertion of a suitable formal power-series ansatz for $\Theta^{\left(JM\right)}$ led to a termination of the infinite series and to a closed and compact inner-product metric

(19) $$\Theta^{\left(JM\right)}\left(\lambda \right)=\exp\left[p^{3}/\left(48\;\lambda^{2}\right)-2\;p\right]$$

(cf. formula Nr. 16 in loc. cit.). In this manner, Jones and Mateo obtained the desirable meaningful physical interpretation of their wrong-sign quartic model in which all of the observable quantities have to be represented by the operators $Q^{\left(JM\right)}$, which have to obey the quasi-Hermiticity constraint (15) in $H_{auxiliary}=L^{2}\left(R\right)$.

A formally analogous constructive NSP recipe can be also applied to the other wrong-sign anharmonic oscillators and/or to their PT—symmetric stationary alternatives. In the Buslaev and Grecchi model [2], in particular, the presence of more complicated components in the interaction also made the Ω—mediated reconstruction of the isospectral self-adjoint Hamiltonian $h^{\left(BG\right)}$ of Equation (10) conceptually fully analogous but, in purely technical terms, perceivably more complicated, with a central role still played by an interchange p↔x mediated by Fourier transformation.

## 4. Quantum Theory in Non-Stationary Dynamical Regime

Briefly, the transition from the conventional, Hermitian version of quantum mechanics (represented in Schrödinger picture [13]) to its non-Hermitian (or, better, quasi-Hermitian) generalization can be characterized as a separation and transfer of the two different roles played by the single textbook Hilbert space $L=H^{\left(T\right)}$ to the two non-equivalent Hilbert spaces, viz., to the “friendly” space $H^{\left(F\right)}$ and to the “physical” space $H^{\left(P\right)}$.

### 4.1. Evolution Equations for States

After one decides to relax the auxiliary technical stationarity assumption and after one admits the manifestly time-dependent Dyson maps Ω=Ω(t) and metrics $\Theta =\Omega^{†}\left(t\right)\;\Omega \left(t\right)=\Theta \left(t\right)$, an enhancement of the flexibility of the formalism becomes accompanied by an increase of the complexity of the theory.

Naturally, the basic idea, i.e., the use of the (this time, time-dependent) Dyson mapping

(20) $$|\psi^{}\left(t\right)\;\;≻\;\;=\Omega \left(t\right)\;|\psi^{}\left(t\right)\rangle \in H^{\left(T\right)}\;,\;\;\;\;\;\;|\psi^{}\left(t\right)\rangle \in H^{\left(F\right)}\;$$

remains unchanged. Nevertheless, its consequences become less straightforward. First of all, an easy observation is that the self-adjoint Hamiltonian $h_{\left(SP\right)}$ or $h_{\left(SP\right)}\left(t\right)$ of the textbooks still becomes replaced by its isospectral “quasi-Hermitian-Hamiltonian” image

(21) $$H_{}\left(t\right)=\Omega_{}^{\left(-1\right)}\left(t\right)\;h_{\left(SP\right)}\left(t\right)\;\Omega_{}\left(t\right)\;$$

acting in $H^{\left(F/P\right)}$ or, more precisely, acting and self-adjoint in $H^{\left(P\right)}$ and acting and non-Hermitian in $H^{\left(F\right)}$ while, in a more appropriate language of review [9], compatible with the constraint

(22) $$H^{†}\left(t\right)\;\Theta \left(t\right)=\Theta^{}\left(t\right)\;H_{}\left(t\right)\;,\;\;\;\;\Theta \left(t\right)=\Omega^{†}\left(t\right)\;\Omega \left(t\right)\;$$

*alias* quasi-Hermitian in $H^{\left(F\right)}$.

On these grounds, it is not too difficult to recall the NIP formalism of papers [17,18] and re-write the SP Schrödinger Equation (11) of the textbooks in its upgraded NIP form in $H^{\left(F\right)}$,

(23) $$i\frac{\partial }{\partial t}\;|\psi^{}\left(t\right)\rangle =G_{}\left(t\right)\;|\psi^{}\left(t\right)\rangle \;,\;\;\;\;G\left(t\right)=H\left(t\right)-\Sigma \left(t\right)$$

where the symbol

(24) $$\Sigma \left(t\right)=i\Omega^{-1}\left(t\right)\;\dot{\Omega }\left(t\right)\;,\;\;\;\;\;\dot{\Omega }\left(t\right)=\frac{d}{dt}\;\Omega \left(t\right)\;$$

denotes the so-called Coriolis force, see also [19] for details.

In the latter review [19], we noticed that in the non-stationary NIP setting the time-dependence of the metric makes its explicit construction too costly. For this reason, we strongly recommended avoiding its use and, whenever possible, to replacing it with the use of the “ketkets”, i.e., of the alternative ket vectors |ψ(t)〉〉=Θ(t)|ψ(t)〉 in $H^{\left(F\right)}$.

From the purely pragmatic point of view, this can lead to important simplifications because one can easily verify that

(25) $$|\psi^{}\left(t\right)\;\;≻\;\;={\left[\Omega^{†}\left(t\right)\right]}^{-1}|\psi \left(t\right)\rangle \;\rangle \in H^{\left(T\right)}\;.$$

The definition of the ketkets implies that they may be defined as solutions of a “second” Schrödinger-like equation in $H^{\left(F\right)}$,

(26) $$i\frac{\partial }{\partial t}\;|\psi \left(t\right)\rangle \;\rangle =G^{†}\left(t\right)\;|\psi \left(t\right)\rangle \;\rangle \;.$$

One can now point out two observations which are important. The first one is that in the NIP framework we can still consider any preselected (and, naturally, quasi-Hermitian) operator (say, Q(t)) representing an observable,

(27) $$Q_{}\left(t\right)=\Omega_{}^{\left(-1\right)}\left(t\right)\;q_{\left(SP\right)}\left(t\right)\;\Omega_{}\left(t\right)\;$$

i.e., quasi-Hermitian,

(28) $$Q_{}^{†}\left(t\right)\;\Theta \left(t\right)=\Theta \left(t\right)\;Q\left(t\right)\;.$$

The second observation is that once we are given Q(t) and use and solve the second Schrödinger-like equation, we are immediately able to evaluate the matrix elements of interest,

(29) $$\langle \;\langle \psi^{}\left(t_{f}\right)\;|Q_{}\left(t_{f}\right)\;|\psi^{}\left(t_{f}\right)\rangle \;$$

i.e., in other words, we can in fact predict the results of the measurements without the explicit construction of the metric.

### 4.2. Physics behind the Equations

Using our present notation we read, in Theorem Nr. 2 of review [5], that “if the time evolution …is unitary and G(t) is an observable, …then the metric …does not depend on time, i.e., there must exist a time-independent operator Θ”. The statement has been based on a tacit assumption that “the time-evolution of the system …is determined by the Schrödinger equation” (i.e., in our present paper, by Equation (23)).

In the preceding subsection we explained that such an assumption is only acceptable in the NSP (i.e., stationary) setting. Indeed, once we decide to admit the time-dependent metrics Θ(t), the ket-vector $|\psi \left(t\right)\rangle \in H^{\left(F\right)}$ does not suffice to specify, by itself, the state of the system in $H^{\left(F\right)}$. For the reasons explained in the related literature [20], one must add also the information about the metric or, equivalently, about the second ket-vector $|\psi \left(t\right)\rangle \;\rangle \in H^{\left(F\right)}$, with its time-evolution controlled by the other, conjugate, independent Schrödingerian Equation (26).

In a more formal language of review [19], a compatibility of the probabilistic predictions (based on formula (29)) with the concept of the description of the state can most easily be achieved when we represent the states with elementary projectors

(30) $$\pi_{\Theta }\left(t\right)=|\psi \left(t\right)\rangle \;\frac{1}{\langle \;\langle \psi (t)|\psi (t)\rangle }\;\langle \;\langle \psi \left(t\right)|$$

i.e., when we decide to work with the concept of a biorthogonal basis [20] and when we make use of the knowledge of *both* the kets |ψ(t)〉 and the (metric-dependent) bras 〈〈ψ(t)|.

In the complete and physical (hypothetical) Hilbert space $H^{\left(P\right)}$, the corresponding twins |ψ(t)≻ and ≺ψ(t)| of the respective kets and bras form, naturally, as the “trivial” Hermitian-conjugate pairs. Nevertheless, once the whole theory is formulated in its mathematically friendliest representation in $H^{\left(F\right)}$, we must work with the full projectors (30) because only these pure-state projectors keep trace of the information about the metric as encoded in 〈〈ψ(t)|, say, via definition (25) [18].

This being said we are prepared to return, once more, to Equation (29). The ultimate necessity of evaluation of the hypothetical textbook matrix elements $≺\;\;\psi^{}\left(t_{f}\right)\left|q_{}\left(t_{f}\right)\right|\psi^{}\left(t_{f}\right)\;\;≻\;\;$ of experimentalist interest is transferred there, via the metric-containing formula $\langle \psi^{}\left(t_{f}\right)|\Theta \left(t_{f}\right)Q_{}\left(t_{f}\right)|\psi^{}\left(t_{f}\right)\rangle$, from the computationally inaccessible Hilbert space $H^{\left(P\right)}$ to its technically preferred alternative $H^{\left(F\right)}$. It is then necessary to keep in mind that the corresponding operators representing the observables (being it, in our notation, $q_{}\left(t\right)$ in $H^{\left(P\right)}$ or $Q_{}\left(t\right)$ in $H^{\left(F\right)}$) are also non-stationary.

As long as the mathematical aspects and consequences of the latter time-dependence of the operators of observables will be outlined in the next subsection, let us now only add that, due to the deeply non-Hermitian nature of these operators (as well as of their energy-representing a special case called Hamiltonian), a truly important advantage is being reached when the model in question admits an explicit construction of the integrals of motion. In the present non-Hermitian setting, in particular, the completion of a meaningful quantum theory is often being perceivably facilitated whenever one manages to construct the so-called Lewis-Riesenfeld invariants (for a more detailed exposition and exemplification of such an efficient trick see, e.g., the recent dedicated paper [21]).

### 4.3. Heisenbergian Evolution Equations for Observables

In our preceding comment, the point was that for the experimental and predictive purposes we do not need to solve the evolution equation controlling the time-dependence of the metric at all. Still, in principle, this equation might still retain a role in methodical considerations (see, e.g., [22]). Thus, it may make sense to write it down for the sake of completeness. Still, even in this setting we have a choice between its Coriolis-force-generated (or, if you wish, Heisenbergian) version

(31) $$i\;\frac{\partial }{\partial t}\;\Theta \left(t\right)=\Theta \left(t\right)\;\Sigma \left(t\right)-\Sigma^{†}\left(T\right)\;\Theta \left(t\right)\;$$

and/or its equivalent Schrödingerian alternative

(32) $$i\;\frac{\partial }{\partial t}\;\Theta \left(t\right)=G^{†}\left(t\right)\;\Theta \left(t\right)-\Theta \left(t\right)\;G\left(t\right)\;.$$

One might only add that a serendipitious merit of the Coriolis-force-controlled approach may be seen in the fact that the knowledge of Σ(t) may be also used in relation

(33) $$i\frac{\partial }{\partial t}\;\Omega^{\left(NIP\right)}\left(t\right)\rangle =\Omega^{\left(NIP\right)}\left(t\right)\;\Sigma^{\left(NIP\right)}\left(t\right)\;,$$

i.e., if needed, for a reconstruction of the time-dependent Dyson map.

In the next step let us now return to definition (27) of a generic observable, and let us differentiate it formally with respect to time. What we obtain is a Heisenbergian evolution equation

(34) $$i\;\frac{\partial }{\partial t}\;Q_{}\left(t\right)=Q\left(t\right)\;\Sigma \left(t\right)-\Sigma_{}\left(t\right)\;Q\left(t\right)+K\left(t\right)\;,\;\;\;\;\;K\left(t\right)=\Omega_{}^{\left(-1\right)}\left(t\right)\;i\;{\dot{q}}_{\left(SP\right)}\left(t\right)\;\Omega_{}\left(t\right)\;.$$

Although such an equation is an immediate parallel of its Hermitian interaction picture (IP) predecessor known in the conventional quantum theory of textbooks, such a general version of the equation is usually considered overcomplicated and rarely used and solved. In practice, such an equation is only considered usable in the quantum systems in which the SP version of the operator remains time-independent, with ${\dot{q}}_{\left(SP\right)}\left(t\right)=0$ and, hence, with K(t)=0.

Sometimes, an exception is tolerated in non-conservative systems in which one may be forced to work, say, with the energy-representing Hamiltonian H(t) for which the operator

$$K^{\left(NIP\right)}\left(t\right)=\Omega_{}^{\left(-1\right)}\left(t\right)\;i\;{\dot{h}}_{\left(SP\right)}\left(t\right)\;\Omega_{}\left(t\right)\;$$

does not vanish. In such a case the only choice is between the Heisenbergian version of the Coriolis-controlled equation

(35) $$i\;\frac{\partial }{\partial t}\;H^{\left(NIP\right)}\left(t\right)=H^{\left(NIP\right)}\left(t\right)\;\Sigma^{\left(NIP\right)}\left(t\right)-\Sigma^{\left(NIP\right)}\left(t\right)\;H^{\left(NIP\right)}\left(t\right)+K^{\left(NIP\right)}\left(t\right)$$

and its equivalent, G(t)—generated alternative

(36) $$i\;\frac{\partial }{\partial t}\;H^{\left(NIP\right)}\left(t\right)=G^{\left(NIP\right)}\left(t\right)\;H^{\left(NIP\right)}\left(t\right)-H^{\left(NIP\right)}\left(t\right)\;G^{\left(NIP\right)}\left(t\right)+K^{\left(NIP\right)}\left(t\right)\;$$

in the derivation of which one takes the advantage of the equal-time commutativity of H(t) with itself.

## 5. Time-Dependent Wrong-Sign Oscillators

### 5.1. The Fring and Tenney Construction

In Fring’s and Tenney’s pioneering application [11] of the NIP approach to non-stationary wrong-sign oscillators, the authors decided to start from a suitable four-parametric ansatz

(37) $$\Omega^{\left(FT\right)}\left(t\right)=e^{\alpha (t)x}e^{\beta \left(t\right)p^{3}+i\gamma \left(t\right)p^{2}+i\delta \left(t\right)p}\;.$$

Such a preselected form of the Dyson map depended on the four real and sufficiently smooth functions α(t),β(t),γ(t), and δ(t) of time (cf. equation Nr. (2.3) in loc. cit.). This immediately led to the Coriolis force

(38) $$\Sigma^{\left(FT\right)}\left(t\right)=ix\dot{\alpha }+i\dot{\beta }p^{3}-\left(3\dot{\alpha }\beta +\dot{\gamma }\right)p^{2}-\left(2i\gamma \dot{\alpha }+\dot{\delta }\right)p-i\delta \dot{\alpha }$$

where the arguments (time) have been dropped, and where the authors marked the partial derivatives with respect to time by an overdot. In a way that was inspired by Jones and Mateo [16], the authors also picked up the Schrödinngerian generator

(39) $$G^{\left(FT\right)}\left(t\right)=p^{2}+\frac{m(t)}{4}z^{2}-\frac{\lambda^{2}\left(t\right)}{16}z^{4}\;,\;\;\;\;z=z\left(x\right)=-2i\sqrt{1+ix}\;,\;\;\;\;x\in R$$

defined in terms of the other two real functions m(t) and γ(t) of time. This enabled them to construct the Hamiltonian operator (i.e., energy operator)

(40) $$H^{\left(FT\right)}\left(t\right)=G^{\left(FT\right)}\left(t\right)+\Sigma^{\left(FT\right)}\left(t\right)$$

as well as the product $\Theta \left(t\right)=\Omega^{†}\left(t\right)\;\Omega \left(t\right)$ and the product $h\left(t\right)=\Omega \left(t\right)\;H\left(t\right)\;\Omega {(}^{-1}t)$. As long as the sum (40) had to be Θ(t)—quasi-Hermitian (i.e., equivalently, as long as the product h(t) had to be Hermitian), the authors just had to satisfy these conditions via a judicious adaptation of the parameters.

The result had the form of definition of all of the four Dyson map “output” functions α(t), β(t) γ(t), and δ(t) in terms of the “input mass” m(t), “input coupling” λ(t), and another real constant $c_{1}$ (cf. equation Nr. (2.11) in loc. cit.). This definition appeared accompanied by another constraint which has been given its final form of definition of mass and coupling in terms of a new unconstrained function σ(t) of time,

(41) $$\lambda^{2}\left(t\right)=\frac{1}{4\sigma^{3}\left(t\right)},\;\;\;\;\;\;\;\;\;m\left(t\right)=\frac{4c_{2}+{\dot{\sigma }}^{2}\left(t\right)-2\sigma \left(t\right)\ddot{\sigma }\left(t\right)}{4\sigma^{2}\left(t\right)}\;.$$

The symbol $c_{2}$ denotes here another free real constant (cf. equation Nr. (2.13) in loc. cit.).

One can conclude that the simultaneous choice of the Schrödingerian generator G(t) and of the Heisenbergian generator Σ(t) led to a feasible construction of the model in which the authors were able to achieve the ultimate goal of the guarantee of isospectrality between the time-dependent pre-selected operator sum H(t)=G(t)+Σ(t) and a (still fairly complicated) self-adjoint operator h(t).

A further, unitary transformation-mediated simplification also appeared to be feasible. Although it still led to a fairly broad multiparameetric family of non-stationary analogues of the Jones and Mateo tilded stationary Hamiltonian (7), further special choices of parameters resulted, finally, in a model exhibiting an almost complete analogy with the Jones and Mateo double-well model (cf. the truly impressive picture Nr. 1 in [11]).

### 5.2. Physical Background

The feasibility of Fring’s and Tenney’s closed-form construction offers a deep insight into the structure of the theory and reopens the further fundamental questions concerning the specification of the physical content of the wrong-sign quantum models or, in general, in the words of review [9], of the way we are “given [a] set of non-Hermitian observables”.

The question has been addressed, from different points of view, in multiple reviews (take just [10,23] for illustration) but the problem may still be considered open. For the purposes of its present analysis, it is sufficient to distinguish between the very broad context of the so-called “non-Hermitian quantum physics” (in which, in a way sampled in monograph [24], the unitarity is not an issue), and the narrower domain of research (of our present interest) which could be called the “quasi-Hermitian quantum physics”.

In the latter setting, naturally, the main emphasis is to be put on the reality of the spectrum. In this sense, a key conceptual role is played by the “implicit” isospectrality relations given by Equation (14) for a generic observable, or by Equation (21) for the instantaneous energy-representing Hamiltonians H(t). The technical and physical background of the quasi-Hermitian theory may be then seen to lie in the replacement of the correct physical Hilbert space $H^{\left(P\right)}$ (which is, by our basic assumption, user-unfriendly) by its representation (using the ad hoc metric operator) in an auxiliary, manifestly unphysical mathematical Hilbert space $H^{\left(F\right)}$.

Formally, this means that given an observable Q(t) or H(t) (or, in an extreme setup, an irreducible set of observables [9] which are, in general, introduced as non-Hermitian in $H^{\left(F\right)}$), the main task is to guarantee the necessary reality of their spectra. In this sense, Fring and Tenney only achieved the goal by “brute force”. A priori, there was really no reason to believe that after their “input” choice of Σ(t) (cf. Equation (38) above) and of G(t) (cf. Equation (39) above) they might eventually succeed in forcing the spectrum of their manifestly non-Hermitian Hamiltonian (40) to be real. That’s why we have returned to the problem of the wrong-sign oscillators in our present paper.

An independent word of warning also comes from paper [25], in which the NSP and NIP formulations of the quasi-Hermitian quantum mechanics were complemented by the introduction of a non-Hermitian Heisenberg picture (NHP). In it, one simply takes the general NIP formalism and sets, irrespectively of any dynamics, $G\left(t\right)=G^{\left(NHP\right)}\left(t\right)\equiv 0$. Naturally, the resulting NHP theoretical framework still admits an arbitrary specification of the unitary quantum dynamics (via a suitable definition of H(t)). Nevertheless, a rather unpleasant surprise was that the formalism only becomes consistent in the stationary case, i.e., for the models using the time-independent metric Θ(t)=Θ(0)=Θ. In this sense, the description of the non-stationary non-Hermitian quantum systems requires, in general the use of the full-fledged NIP formalism. The applicability of both of its simplified (viz., NSP and NHP) alternatives remains restricted to the stationary systems.

### 5.3. Alternative, Physics-Motivated NIP Constructions

In the non-stationary models which are non-Hermitian in $H^{\left(F\right)}$, one should resist the temptation of choosing the “false” Schrödingerian Hamiltonian G(t) or the Heisenbergian Hamiltonian Σ(t) in advance. Indeed, besides a few mathematical but more or less purely formal advantages of such an option (as discussed, more thoroughly, in [22,26,27,28] or in review [19]), the phenomenological role of these operators is secondary. In the manner explained in the older review [9] the initial physical information about quantum dynamics is almost exclusively carried by the operators of observables.

In spite of a certain mathematical user-friendliness of the choices of G(t) and/or Σ(t), one can only speak about the closed-system *unitary* evolution when one manages to guarantee the reality of the spectrum of the observable of interest and, in particular, of H(t), i.e., of *the sum* of G(t) and Σ(t). In a way illustrated by an explicitly solvable example in [29], a consistent NIP description of unitary systems can be obtained even when the spectra of G(t) and/or of Σ(t) happen to be complex. Additionally, in the quasi-Hermitian quantum mechanics of the wrong-sign oscillators these spectra cannot have any immediate physical meaning.

An intuitive clarification of the apparent paradox is not too difficult. It is sufficient to imagine that from the point of view of the predictions of the theory (mediated by the matrix elements (29)) the reality of the spectrum of the observable Hamiltonian H(t) is caused by a mutual fine-tuned cancellation of the complexities generated by the two “false” Hamiltonians G(t) and Σ(t).

Naturally, whenever one starts from an initial choice of G(t) and/or Σ(t), a guarantee of the latter cancelation need not be easy. This is well documented even by Fring’s and Tenney’s paper [11] itself. In it, the authors revealed that in the massless case with m(t)=0 one has to fix

$$\sigma^{\left[0\right]}\left(t\right)=\kappa_{0}+\kappa_{1}t+\kappa_{2}t^{2}\;,\;\;\;\;c_{2}^{\left[0\right]}=\kappa_{1}\kappa_{3}-\kappa_{2}^{2}/4\;$$

due to the second relation in (41). Nevertheless, the Jones and Mateo time-independent solutions of paper [16] still cannot be reproduced in any ad hoc limit because, citing Fring’s and Tenney’s own words, “the constraints for γ and δ [in ansatz (37) above] are different from those reported” [i.e., reported in equation Nr. (2.11) of loc. cit.] so that “there is no time-dependent solution corresponding to that choice” [11].

From our present point of view the resolution of the “massless-case” puzzle would lie in the replacement of the false non-stationary Hamiltonian G(t) (i.e., of ansatz (39)) by an alternative definition

(42) $$H^{\left(NJM\right)}\left(t\right)=-\frac{d^{2}}{dz^{2}}-g\left(t\right)\;z^{4}\;$$

of an analogous non-stationary observable JM-like Hamiltonian living on the same complex contour (6) of the “coordinate” *z* as its stationary predecessor (5).

In such an alternative setting one immediately sees, first of all, that the innovated, non-stationary wrong-sign NJM oscillator will still possess the real spectrum. In other words, the brute-force introduction of the time-dependence cannot destroy the observability property of Hamiltonian (42). The guarantee of its spectral equivalence with its self-adjoint avatar

(43) $${\tilde{h}}^{\left(NJM\right)}\left(t\right)=-\frac{d^{2}}{dy^{2}}-2\;\sqrt{g(t)}\;y+4\;g\left(t\right)\;y^{4}\;.$$

as prescribed by Equation (7) is purely algebraic. The newly introduced variability with time does not play any role. One reveals that in many specific models and applications the introduction of a nontrivial and sufficiently general time-dependence *directly into the observables* (like, e.g., into H(t)) might be not only warmly phenomenologically welcome (say, in the manybody quantum physics [30]) but also, in the strictly mathematical sense, simpler than the (hardly phenomenologically motivated) choice of G(t) and/or Σ(t).

From such a perspective, many transitions to non-stationary dynamical regime might very easily be realized by the introduction of a more or less arbitrary time-dependence in the original parameters of stationary dynamics which specify, say, directly the observable Hamiltonian H(t). In comparison with the constructions based on the input knowledge of Ω(t) and G(t) the alternative input knowledge of the mere H(t) might enable one to reconstruct, in the manner used by Jones and Mateo [16], the admissible forms of Ω(t) via the direct factorization of the metric, i.e., more easily, in principle at least.

In the specific and most elementary Jones and Mateo one-parametric and stationary wrong-sign oscillator (5), the elementary replacement of the positive constant $\lambda^{2}$ by a suitable positive and not unreasonable time-dependent function g(t) immediately leads to the explicit form of the non-stationary Dyson map

(44) $$\Omega^{\left(NJM\right)}\left(t\right)=\exp\left[p^{3}/\left[96\;g\left(t\right)\right]-p\right]$$

(cf. also the corresponding exact form of the operator of metric as given in Equation (19) above). In light of this observation, the core of our present message can be now formulated as a statement that once one knows the details of the construction of an arbitrary NSP model, the knowledge of the stationary version (14) of the isospectrality between the different representations of an observable in question could often be most easily extended to its non-stationary NIP version (i.e., say, to relation (21) between Hamiltonians).

The non-stationarity of the operators only enters the game when one further proceeds to the definitions of the Coriolis force Σ(t) (using the elementary definition (24), which is still an entirely straightforward mathematical operation) and of the related Schrödingerian generator G(t)=H(t)−Σ(t). In other words, the price for the easiness of construction of the non-stationary JM-type Dyson maps (sampled by Equation (44)) is to be paid, in applications, by the more complicated forms of the Heisenberg and Schrödinger equations of the general methodical Section 4 above.

## 6. Conclusions

In the literature, multiple ordinary differential non-Hermitian models have been studied and described in the stationary NSP framework (for illustration see, e.g., the Lévai’s chapter in monograph [10]). In parallel, it seems much more difficult to overcome the technical obstacles encountered in the more general non-stationary NIP setting. For this reason, our attention has been attracted by one of very few non-stationary and non-Hermitian wrong-sign models, the exact solvability of which has been achieved by Fring and Tenney [11].

The appeal of their model as well as of their method may be found rooted in mathematics as well as in physics. In both of these contexts the puzzling “wrong-sign“ quartic-oscillator potentials $V\left(x\right)=-\lambda^{2}\;x^{4}+O\left(x^{3}\right)$ emerged as a byproduct of analysis of the standard self-adjoint anharmonic oscillator Hamiltonian (1). The history of the anomaly has briefly been recollected by Buslaev with Grecchi [2]. Their careful mathematical study of the realistic and centrally symmetric d—dimensional model (1) revealed that at a fixed angular momentum quantum number *ℓ* and its bound-state spectrum of energies $\left\{E_{n,\ell }\left(\lambda \right)\right\}$ appears to coincide with the spectrum of another, complex and non-Hermitian and, seemingly, far from realistic Hamiltonian-like differential operator (2).

In applied quantum physics, the references to the d—dimensional oscillators of Equation (1) abound. They range from the pragmatic constructions of bound states in atomic and molecular systems [1] up to the purely methodical considerations with relevance in field theory [31,32]. Their study also became popular due to its mathematical user-friendliness. One of the best known features of the model is that it offers a sufficiently feasible tool for the tests and applications of perturbation theory [33,34,35] as well as of the non-Hermitian versions of quantum mechanics [36].

In the latter case, one really has to be impressed by Buslaev’s and Grecchi’s isospectrality results as mentioned in the Introduction. Even more so because the tilded operator (2) itself can be perceived as a specific quantum Hamiltonian in which just the potential is complex and asymptotically decreasing. For such a reason the discreteness, reality, and boundedness of the spectrum of operator (2) may look counterintuitive.

With time, the latter complex differential Hamiltonian-like operator appeared to belong to a broader class with similar properties. The reality of eigenvalues has been shown to survive the omission of the asymptotically dominant quartic term $-\lambda^{2}\;x^{4}$ from interaction in Equation (2) (see, e.g., the detailed studies of the resulting imaginary cubic anharmonic oscillators in [36,37,38]). The manifestly non-Hermitian models with real spectra as sampled by Equation (2) were returned to the mainstream of quantum physics.

In our present paper we paid attention to several formal as well as interpretative challenges encountered after transition of the non-stationary version of the theory. After such a transition the necessity of making the manifestly non-Hermitian model compatible with the standard probabilistic interpretation and postulates of quantum mechanics appears particularly difficult. We believe that we managed to clarify a few misunderstandings which emerged recently in such a context. In this sense, our main message is that once one sufficiently clearly distinguishes between the observable and non-observable operators (which could be all called Hamiltonians), the NIP-based treatment of the wrong-sign quartic oscillators becomes fully acceptable and void of counterintuitive features and paradoxes.

## Data Availability

Not applicable.
